# Supplementary material for: The Impact of Phyllostachys heterocyclas Expansion on the Phylogenetic Diversity and Community Assembly of Subtropical Forest
Source: Plants (Basel). 2025 Oct 21;14(20):3231. doi: 10.3390/plants14203231 (PMC12566707; doi:10.3390/plants14203231)
Supplement: Supplementary file 1 [file plants-14-03231-s001.zip › Figure S1 Phylogenetic tree of the entire plant community.pdf]

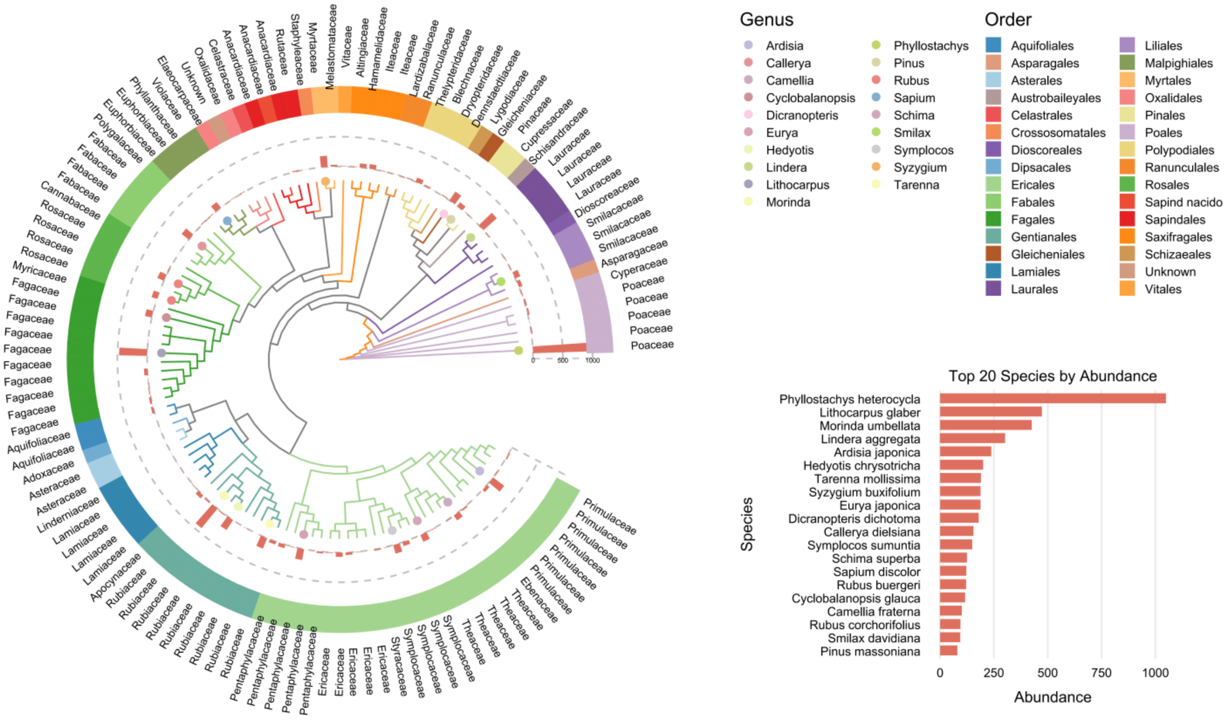

Figure S1. Phylogenetic tree of the entire plant community (branch colors indicate different orders; outer labels denote families; the bar chart overlays show the top-20 species by overall importance; colored nodes highlight the genera of those top-20 species)
