# Supplementary material for: The Impact of Phyllostachys heterocyclas Expansion on the Phylogenetic Diversity and Community Assembly of Subtropical Forest
Source: Plants (Basel). 2025 Oct 21;14(20):3231. doi: 10.3390/plants14203231 (PMC12566707; doi:10.3390/plants14203231)
Supplement: Supplementary file 1 [file plants-14-03231-s001.zip › Table S1 Distribution of plant species in the tree, shrub, and herb layers along the Moso bamboo expansion gradient.pdf]

Table S1. Distribution of plant species in the tree, shrub, and herb layers along the Moso bamboo expansion gradient

Layer	Species	Dominance(%)	Density(%)	Frequency(%)	Iv(%)	Treat
	Phyllostachys heterocycla	1	1	1	1	MB
	Elaeocarpus chinensis	0.03	0.41	2.17	0.87	HM
	Vaccinium trichocladum	0.06	1.22	2.17	1.15	HM
	Ternstroemia gymnanthera	0.05	0.61	2.17	0.95	HM
	Styrax dasyanthus	0.02	0.20	2.17	0.80	HM
	Symplocos sumuntia	0.05	0.61	4.35	1.67	HM
	Schima superba	6.42	4.49	6.52	5.81	HM
	Toxicodendron sylvestre	0.02	0.20	2.17	0.80	HM
	Adinandra millettii	0.21	0.61	4.35	1.72	HM
	Liquidambar formosana	2.80	1.02	4.35	2.72	HM
	Eurya japonica	0.20	1.43	6.52	2.72	HM
	Lithocarpus glaber	9.01	8.78	8.70	8.83	HM
	Gardenia jasminoides	0.02	0.20	2.17	0.80	HM
	Loropetalum chinense	0.16	1.02	4.35	1.84	HM
	Camellia fraterna	0.01	0.41	2.17	0.86	HM
	Phyllostachys heterocycla	61.70	68.37	8.70	46.26	HM
	Quercus fabri	0.02	0.20	2.17	0.80	HM
	Eurya nitida	0.07	0.61	4.35	1.68	HM
	Castanopsis sclerophylla	1.71	1.84	6.52	3.36	HM
	Syzygium buxifolium	0.01	0.20	2.17	0.80	HM
	Cyclobalanopsis glauca	4.47	4.69	8.70	5.95	HM
	Pinus massoniana	12.86	2.24	8.70	7.93	HM
Tree	*Rhododendron ovatum*	0.04	0.41	2.17	0.87	HM
	Dalbergia hupeana	0.05	0.20	2.17	0.81	HM
	Quercus phillyraeoides	0.06	0.17	1.59	0.61	LM
	Vaccinium trichocladum	0.08	1.01	3.17	1.42	LM
	Vaccinium bracteatum	0.05	0.17	1.59	0.60	LM
	Ternstroemia gymnanthera	0.42	2.85	1.59	1.62	LM
	Styrax dasyanthus	0.17	1.17	4.76	2.04	LM
	Camellia cuspidata	0.10	1.51	1.59	1.07	LM
	Symplocos sumuntia	0.36	1.68	4.76	2.27	LM
	Schima superba	8.81	5.54	4.76	6.37	LM
	Cunninghamia lanceolata	0.16	0.17	1.59	0.64	LM
	Adinandra millettii	0.13	0.84	3.17	1.38	LM
	Liquidambar formosana	0.72	0.34	1.59	0.88	LM
	Eurya japonica	0.55	5.87	4.76	3.73	LM
	Lithocarpus glaber	27.57	20.97	6.35	18.30	LM
	Loropetalum chinense	0.26	1.68	4.76	2.23	LM
	Camellia fraterna	0.18	2.52	3.17	1.96	LM
	Phyllostachys heterocycla	29.76	30.03	6.35	22.05	LM
	Quercus fabri	0.02	0.17	1.59	0.59	LM
	Raphiolepis indica	0.01	0.17	1.59	0.59	LM
	Castanopsis carlesii	1.36	1.01	1.59	1.32	LM
	Eurya nitida	0.02	0.34	1.59	0.65	LM
	Symplocos stellaris	0.04	0.34	3.17	1.18	LM

	Castanopsis sclerophylla	4.74	5.70	6.35	5.60	LM
	Syzygium buxifolium	0.16	1.51	4.76	2.14	LM
	Diospyros kaki	0.23	0.50	3.17	1.30	LM
	Cyclobalanopsis glauca	9.02	8.05	6.35	7.81	LM
	Pinus massoniana	14.67	3.36	6.35	8.13	LM
	Rhododendron ovatum	0.06	1.01	3.17	1.41	LM
	Dalbergia hupeana	0.26	0.67	3.17	1.37	LM
	Itea chinensis	0.04	0.67	1.59	0.77	LM
	Elaeocarpus chinensis	0.05	0.21	1.56	0.61	BF
	Quercus phillyraeoides	0.63	1.06	1.56	1.09	BF
	Lindera aggregata	0.09	2.12	4.69	2.30	BF
	Ilex chinensis	0.07	0.21	1.56	0.62	BF
	Vaccinium trichocladum	0.64	4.88	6.25	3.92	BF
	Ternstroemia gymnanthera	1.27	4.46	3.13	2.95	BF
	Styrax dasyanthus	0.08	1.27	3.13	1.49	BF
	Elaeocarpus sylvestris	0.10	0.21	1.56	0.62	BF
	Symplocos sumuntia	0.24	1.91	4.69	2.28	BF
	Litsea cubeba	0.01	0.21	1.56	0.59	BF
	Schima superba	14.62	8.70	3.13	8.82	BF
	Rhododendron simsii	0.04	0.64	1.56	0.75	BF
	Adinandra millettii	0.10	0.64	1.56	0.77	BF
	Myrica rubra	1.42	0.64	3.13	1.73	BF
	Eurya japonica	1.14	7.86	6.25	5.08	BF
	Lithocarpus glaber	30.02	25.69	6.25	20.65	BF
	Loropetalum chinense	0.45	2.76	4.69	2.63	BF
	Camellia fraterna	0.16	2.34	4.69	2.40	BF
	Symplocos stellaris	0.50	1.49	4.69	2.22	BF
	Castanopsis sclerophylla	0.73	1.49	6.25	2.82	BF
	Syzygium buxifolium	1.10	7.64	4.69	4.48	BF
	Diospyros kaki	0.90	1.49	4.69	2.36	BF
	Cyclobalanopsis glauca	1.51	3.82	4.69	3.34	BF
	Pinus massoniana	43.16	10.62	6.25	20.01	BF
	Rhododendron ovatum	0.87	5.94	3.13	3.31	BF
	Itea chinensis	0.10	1.70	4.69	2.16	BF
**Shrub**	*Elaeocarpus chinensis*	0.41	0.56	2.14	1.04	MB
	Lindera aggregata	8.26	2.97	2.86	4.70	MB
	Phyllostachys sulphurea	1.33	0.24	0.71	0.76	MB
	Vaccinium trichocladum	0.06	0.08	0.71	0.29	MB
	Choerospondias axillaris	1.57	1.44	2.14	1.72	MB
	Ternstroemia gymnanthera	0.17	0.40	0.71	0.43	MB
	Celtis tetrandra	0.39	0.08	0.71	0.39	MB
	Smilax glabra	0.10	2.09	2.86	1.68	MB
	Styrax dasyanthus	0.45	0.16	0.71	0.44	MB
	Clerodendrum cyrtophyllum	0.10	0.08	0.71	0.30	MB
	Viburnum erosum	0.75	0.24	2.14	1.05	MB
	Embelia vestita	0.13	0.48	2.14	0.92	MB
	Rubus buergeri	1.99	8.51	2.86	4.45	MB
	Smilax davidiana	1.81	2.57	2.86	2.41	MB
	Sapium discolor	0.79	7.30	2.86	3.65	MB

Symplocos sumuntia	4.91	1.93	2.86	3.23	MB
Rubus corchorifolius	2.67	7.54	2.14	4.12	MB
Itea omeiensis	2.88	1.04	2.86	2.26	MB
Schima superba	5.56	1.77	0.71	2.68	MB
Indigofera tinctoria	0.12	0.16	0.71	0.33	MB
Cunninghamia lanceolata	3.08	0.32	1.43	1.61	MB
Liquidambar formosana	0.88	0.24	0.71	0.61	MB
Eurya japonica	13.28	4.49	2.86	6.88	MB
Lithocarpus glaber	1.11	1.28	1.43	1.27	MB
Gardenia jasminoides	1.54	1.52	2.86	1.97	MB
Quercus aliena	0.58	0.16	0.71	0.48	MB
Loropetalum chinense	4.29	1.44	2.86	2.86	MB
Ilex pubescens	1.56	1.12	2.86	1.85	MB
Camellia fraterna	1.07	0.88	2.14	1.36	MB
Phyllostachys heterocycla	4.41	0.56	1.43	2.13	MB
Adina pilulifera	0.10	0.08	0.71	0.30	MB
Coptosapelta diffusa	0.20	0.40	0.71	0.44	MB
Nekemias cantoniensis	0.39	0.64	2.14	1.06	MB
Mussaenda pubescens	0.16	0.48	0.71	0.45	MB
Mallotus apelta	0.99	0.88	1.43	1.10	MB
Tarenna mollissima	5.05	3.85	2.86	3.92	MB
Rhus chinensis	0.10	0.16	0.71	0.32	MB
Vaccinium carlesii	0.33	0.32	2.14	0.93	MB
Raphiolepis indica	0.11	0.16	0.71	0.33	MB
Zanthoxylum armatum	0.07	0.16	0.71	0.31	MB
Indocalamus tessellatus	0.87	0.08	0.71	0.56	MB
Ardisia japonica	5.79	17.66	2.86	8.77	MB
Lindera erythrocarpa	0.96	0.80	2.14	1.30	MB
Callicarpa rubella	0.31	0.48	1.43	0.74	MB
Illicium dunnianum	0.62	0.16	1.43	0.74	MB
Trachelospermum jasminoides	0.62	5.06	2.14	2.61	MB
Morinda umbellata	0.96	5.94	2.86	3.25	MB
Symplocos stellaris	0.72	0.32	1.43	0.82	MB
Paederia cruddasiana	0.85	1.52	2.86	1.74	MB
Camellia sinensis	3.41	0.96	2.14	2.17	MB
Syzygium buxifolium	3.01	0.64	2.14	1.93	MB
Celastrus aculeatus	0.06	0.08	0.71	0.29	MB
Diospyros kaki	1.63	1.20	2.14	1.66	MB
Castanopsis chinensis	3.75	0.56	1.43	1.92	MB
Hylodesmum podocarpum	0.03	0.08	0.71	0.28	MB
Cyclobalanopsis glauca	1.16	0.48	1.43	1.02	MB
Callerya dielsiana	1.47	5.14	2.86	3.15	MB
Lindera aggregata	7.03	9.62	3.20	6.62	HM
Ardisia brevicaulis	0.30	0.57	1.60	0.82	HM
Holboellia fargesii	0.02	0.11	0.80	0.31	HM
Symplocos lancifolia	0.25	0.57	1.60	0.81	HM
Vaccinium trichocladum	1.35	0.34	0.80	0.83	HM
Clematis henryi	0.07	1.37	1.60	1.01	HM
Ternstroemia gymnanthera	1.28	0.69	0.80	0.92	HM

Smilax glabra	0.17	1.49	3.20	1.62	HM
Styrax dasyanthus	0.93	0.57	2.40	1.30	HM
Clerodendrum cyrtophyllum	0.22	0.11	0.80	0.38	HM
Viburnum erosum	0.16	0.11	0.80	0.36	HM
Embelia vestita	0.81	1.95	2.40	1.72	HM
Rubus buergeri	0.40	1.26	1.60	1.09	HM
Smilax davidiana	1.44	2.52	3.20	2.39	HM
Camellia cuspidata	2.93	0.34	0.80	1.36	HM
Sapium discolor	0.27	2.06	1.60	1.31	HM
Symplocos sumuntia	8.80	4.58	3.20	5.53	HM
Ardisia lindleyana	0.07	0.11	0.80	0.33	HM
Itea omeiensis	0.77	1.60	2.40	1.59	HM
Schima superba	0.36	0.23	0.80	0.46	HM
Ardisia crenata	0.04	0.23	0.80	0.36	HM
Cunninghamia lanceolata	0.88	0.69	0.80	0.79	HM
Rhododendron simsii	0.16	1.03	0.80	0.66	HM
Eurya japonica	7.07	4.01	3.20	4.76	HM
Lithocarpus glaber	11.06	8.59	3.20	7.62	HM
Gardenia jasminoides	2.91	1.03	2.40	2.11	HM
Castanea mollissima	1.46	0.11	0.80	0.79	HM
Castanopsis fargesii	0.26	0.34	1.60	0.73	HM
Loropetalum chinense	2.70	1.72	2.40	2.27	HM
Ilex pubescens	5.19	1.60	3.20	3.33	HM
Camellia fraterna	2.78	1.83	2.40	2.34	HM
Coptosapelta diffusa	0.39	0.57	2.40	1.12	HM
Diplospora dubia	0.16	0.11	0.80	0.36	HM
Mallotus apelta	0.02	0.11	0.80	0.31	HM
Tarenna mollissima	7.26	8.25	3.20	6.24	HM
Rhus chinensis	0.07	0.11	0.80	0.33	HM
Vaccinium carlesii	0.29	0.11	0.80	0.40	HM
Raphiolepis indica	0.15	1.03	1.60	0.93	HM
Indocalamus tessellatus	5.08	1.26	3.20	3.18	HM
Ardisia japonica	0.63	1.60	1.60	1.28	HM
Eurya nitida	0.83	0.80	2.40	1.34	HM
Trachelospermum jasminoides	0.09	0.46	1.60	0.72	HM
Morinda umbellata	3.02	17.18	3.20	7.80	HM
Symplocos stellaris	4.26	1.95	3.20	3.14	HM
Castanopsis sclerophylla	0.02	0.11	0.80	0.31	HM
Symplocos anomala	0.11	0.11	0.80	0.34	HM
Syzygium buxifolium	7.59	4.81	3.20	5.20	HM
Diospyros kaki	0.34	0.34	0.80	0.49	HM
Rubus reflexus	0.22	0.23	0.80	0.42	HM
Castanopsis chinensis	0.96	0.69	1.60	1.08	HM
Cyclobalanopsis glauca	0.45	0.69	2.40	1.18	HM
Callerya dielsiana	1.60	5.50	3.20	3.43	HM
Smilax lanceifolia	0.08	0.23	0.80	0.37	HM
Rhododendron ovatum	2.32	1.95	3.20	2.49	HM
Dalbergia hupeana	1.94	0.34	0.80	1.03	HM
Lindera aggregata	11.63	13.32	3.88	9.61	LM

Ardisia brevicaulis	0.27	0.88	2.91	1.35	LM
Holboellia fargesii	0.02	0.15	0.97	0.38	LM
Ternstroemia gymnanthera	1.80	0.88	1.94	1.54	LM
Smilax glabra	0.14	1.90	3.88	1.98	LM
Styrax dasyanthus	0.14	0.15	0.97	0.42	LM
Clerodendrum cyrtophyllum	0.06	0.15	0.97	0.39	LM
Embelia vestita	0.20	1.02	3.88	1.70	LM
Rubus buergeri	0.10	0.44	0.97	0.50	LM
Smilax davidiana	0.67	2.05	2.91	1.88	LM
Camellia cuspidata	1.26	0.29	1.94	1.16	LM
Sapium discolor	0.09	1.76	1.94	1.26	LM
Symplocos sumuntia	11.07	4.10	3.88	6.35	LM
Itea omeiensis	9.22	5.42	3.88	6.17	LM
Antidesma japonicum	1.10	0.59	1.94	1.21	LM
Schima superba	0.14	0.15	0.97	0.42	LM
Ardisia crenata	0.41	1.32	0.97	0.90	LM
Liquidambar formosana	0.06	0.15	0.97	0.39	LM
Eurya japonica	3.93	1.17	3.88	2.99	LM
Lithocarpus glaber	10.70	7.76	3.88	7.45	LM
Gardenia jasminoides	1.48	1.90	2.91	2.10	LM
Castanopsis fargesii	0.20	0.29	1.94	0.81	LM
Eurya muricata	1.23	0.15	0.97	0.78	LM
Loropetalum chinense	0.57	0.88	0.97	0.81	LM
Ilex pubescens	1.77	0.88	1.94	1.53	LM
Camellia fraterna	7.53	5.12	3.88	5.51	LM
Adina pilulifera	0.82	0.59	0.97	0.79	LM
Coptosapelta diffusa	0.14	0.59	1.94	0.89	LM
Tarenna mollissima	8.58	7.17	3.88	6.55	LM
Staphylea bumalda	0.19	0.15	0.97	0.43	LM
Vaccinium carlesii	0.98	0.44	0.97	0.80	LM
Raphiolepis indica	0.03	0.29	0.97	0.43	LM
Indocalamus tessellatus	0.45	0.44	1.94	0.94	LM
Castanopsis carlesii	4.34	2.05	1.94	2.78	LM
Ardisia japonica	0.10	0.44	0.97	0.50	LM
Eurya nitida	2.39	1.46	2.91	2.26	LM
Morinda umbellata	3.57	15.96	3.88	7.80	LM
Symplocos stellaris	4.79	2.49	2.91	3.40	LM
Castanopsis sclerophylla	0.43	0.88	0.97	0.76	LM
Syzygium buxifolium	1.60	5.86	3.88	3.78	LM
Castanopsis chinensis	1.35	0.44	0.97	0.92	LM
Cyclobalanopsis glauca	1.07	1.32	2.91	1.77	LM
Callerya dielsiana	1.31	4.83	2.91	3.02	LM
Smilax lanceifolia	0.12	0.59	0.97	0.56	LM
Rhododendron ovatum	1.96	1.17	2.91	2.01	LM
Lindera aggregata	19.88	14.01	3.88	12.59	BF
Ardisia brevicaulis	0.68	2.10	2.91	1.90	BF
Holboellia fargesii	0.24	1.05	1.94	1.08	BF
Vaccinium trichocladum	1.94	0.53	1.94	1.47	BF
Ternstroemia gymnanthera	0.41	0.70	1.94	1.02	BF

	Smilax glabra	0.10	1.58	3.88	1.85	BF
	Styrax dasyanthus	0.01	0.18	0.97	0.39	BF
	Embelia vestita	0.92	4.73	1.94	2.53	BF
	Smilax davidiana	1.99	4.38	3.88	3.42	BF
	Camellia cuspidata	0.10	0.35	1.94	0.80	BF
	Symplocos sumuntia	10.11	6.13	3.88	6.71	BF
	Itea omeiensis	3.20	2.28	3.88	3.12	BF
	Antidesma japonicum	0.12	0.18	0.97	0.42	BF
	Litsea pungens	0.56	0.18	0.97	0.57	BF
	Schima superba	1.65	0.53	1.94	1.37	BF
	Ardisia crenata	0.35	1.05	2.91	1.44	BF
	Eurya japonica	2.50	1.58	2.91	2.33	BF
	Lithocarpus glaber	4.27	6.83	3.88	4.99	BF
	Gardenia jasminoides	0.47	1.58	3.88	1.98	BF
	Castanopsis fargesii	0.20	0.70	1.94	0.95	BF
	Eurya muricata	0.21	0.18	0.97	0.45	BF
	Loropetalum chinense	0.11	0.35	1.94	0.80	BF
	Ilex pubescens	0.66	0.70	2.91	1.42	BF
	Camellia fraterna	3.59	1.75	2.91	2.75	BF
	Camellia oleifera	0.03	0.18	0.97	0.39	BF
	Coptosapelta diffusa	0.41	1.75	2.91	1.69	BF
	Tarenna mollissima	5.56	3.50	3.88	4.32	BF
	Vaccinium carlesii	0.03	0.18	0.97	0.39	BF
	Raphiolepis indica	0.07	0.70	1.94	0.90	BF
	Indocalamus tessellatus	0.42	0.88	0.97	0.75	BF
	Castanopsis carlesii	5.85	2.45	1.94	3.41	BF
	Eurya nitida	2.69	1.23	2.91	2.28	BF
	Morinda umbellata	1.77	16.11	3.88	7.25	BF
	Symplocos stellaris	9.68	3.85	2.91	5.48	BF
	Symplocos anomala	3.92	1.23	0.97	2.04	BF
	Syzygium buxifolium	4.49	8.93	3.88	5.77	BF
	Cyclobalanopsis glauca	0.75	1.05	1.94	1.25	BF
	Callerya dielsiana	0.26	1.58	3.88	1.91	BF
	Smilax lanceifolia	0.13	0.88	1.94	0.98	BF
	Rhododendron ovatum	9.65	1.93	2.91	4.83	BF
**Herb**	*Carex chinensis*	7.07	3.70	5.56	5.44	MB
	Melastoma dodecandrum	0.02	6.16	1.85	2.68	MB
	Eupatorium chinense	13.03	0.62	3.70	5.78	MB
	Mosla dianthera	0.47	0.82	1.85	1.05	MB
	Liriope spicata	9.77	3.29	7.41	6.82	MB
	Lysimachia patungensis	0.23	0.82	1.85	0.97	MB
	Dioscorea japonica	7.91	2.05	5.56	5.17	MB
	Lysimachia fortunei	1.40	0.21	1.85	1.15	MB
	Ainsliaea fragrans	4.19	0.82	3.70	2.90	MB
	Cyclosorus interruptus	2.42	3.49	3.70	3.20	MB
	Lygodium japonicum	3.72	3.49	3.70	3.64	MB
	Lophatherum gracile	2.14	1.23	3.70	2.36	MB
	Woodwardia japonica	12.33	8.62	7.41	9.46	MB
	Polygala hongkongensis	4.84	3.29	7.41	5.18	MB

Viola violacea	1.86	2.05	5.56	3.16	MB
Miscanthus sinensis	3.26	0.82	1.85	1.98	MB
Dicranopteris dichotoma	7.03	11.91	7.41	8.78	MB
Pteridium aquilinum	1.86	0.82	1.85	1.51	MB
Oxalis corniculata	0.47	0.41	1.85	0.91	MB
Hedyotis chrysotricha	0.88	39.63	7.41	15.97	MB
Hylodesmum podocarpum	0.47	0.41	1.85	0.91	MB
Lindernia anagallis	0.84	0.62	1.85	1.10	MB
Scutellaria indica	2.14	2.26	3.70	2.70	MB
Dryopteris fuscipes	11.64	2.46	7.41	7.17	MB
Lophatherum gracile	3.89	3.33	7.69	4.97	HM
Woodwardia japonica	56.75	40.00	30.77	42.51	HM
Miscanthus sinensis	3.89	1.67	7.69	4.42	HM
Dicranopteris dichotoma	15.56	35.00	23.08	24.55	HM
Hedyotis chrysotricha	0.39	6.67	7.69	4.92	HM
Dryopteris fuscipes	19.53	13.33	23.08	18.65	HM
Carex chinensis	26.64	21.05	22.22	23.31	LM
Lophatherum gracile	26.73	47.37	22.22	32.11	LM
Woodwardia japonica	26.64	10.53	22.22	19.80	LM
Dicranopteris dichotoma	8.88	7.89	11.11	9.30	LM
Hedyotis chrysotricha	2.22	7.89	11.11	7.08	LM
Dryopteris fuscipes	8.88	5.26	11.11	8.42	LM
Carex chinensis	13.18	1.82	11.11	8.70	BF
Lophatherum gracile	7.69	3.64	22.22	11.18	BF
Woodwardia japonica	22.00	6.36	22.22	16.86	BF
Dicranopteris dichotoma	57.14	88.18	44.44	63.25	BF
