# Supplementary material for: The Impact of Phyllostachys heterocyclas Expansion on the Phylogenetic Diversity and Community Assembly of Subtropical Forest
Source: Plants (Basel). 2025 Oct 21;14(20):3231. doi: 10.3390/plants14203231 (PMC12566707; doi:10.3390/plants14203231)
Supplement: Supplementary file 1 [file plants-14-03231-s001.zip › Table S2 Species-level phylogenetically weighted Resistance Index (RI) values for tree, shrub, and herb layers.pdf]

Table S2. Species-level phylogenetically weighted Resistance Index (RI) values for tree, shrub, and herb layers

Layer	species	β	MPD	RI	RI-Z	Class_phylo
Tree	*Cyclobalanopsis_glauca*	0.040	271.516	1.46E-04	1.454	Resistant
	Adinandra_millettii	0.014	271.516	5.33E-05	0.951	Neutral
	Castanopsis_sclerophylla	0.008	271.516	2.99E-05	0.824	Neutral
	Symplocos_sumuntia	-0.009	271.516	-3.40E-05	0.476	Neutral
	Styrax_dasyanthus	-0.010	271.516	-3.87E-05	0.451	Neutral
	Loropetalum_chinense	-0.012	271.516	-4.41E-05	0.421	Neutral
	Camellia_fraterna	-0.023	271.516	-8.54E-05	0.196	Neutral
	Ternstroemia_gymnanthera	-0.030	271.516	-1.12E-04	0.052	Neutral
	Eurya_japonica	-0.036	271.516	-1.32E-04	-0.057	Neutral
	Rhododendron_ovatum	-0.037	271.516	-1.36E-04	-0.079	Neutral
	Vaccinium_trichocladum	-0.042	271.516	-1.55E-04	-0.181	Neutral
	Schima_superba	-0.046	271.516	-1.68E-04	-0.252	Neutral
	Syzygium_buxifolium	-0.056	271.516	-2.05E-04	-0.456	Neutral
	Pinus_massoniana	-0.183	650.100	-2.81E-04	-0.870	Neutral
	Lithocarpus_glaber	-0.179	271.516	-6.60E-04	-2.930	Susceptible
Shrub	*Indocalamus_tessellatus*	0.002	3.883	6.33E-04	4.169	Resistant
	Ardisia_japonica	0.114	271.516	4.20E-04	2.752	Resistant
	Rubus_buergeri	0.054	271.516	2.01E-04	1.295	Resistant
	Eurya_japonica	0.046	271.516	1.68E-04	1.080	Resistant
	Sapium_discolor	0.033	271.516	1.21E-04	0.769	Neutral
	Loropetalum_chinense	0.021	271.516	7.90E-05	0.488	Neutral
	Castanopsis_chinensis	0.014	271.516	5.06E-05	0.299	Neutral
	Callerya_dielsiana	0.012	271.516	4.27E-05	0.246	Neutral
	Camellia_cuspidata	0.009	271.516	3.14E-05	0.171	Neutral
	Schima_superba	0.007	271.516	2.63E-05	0.137	Neutral
	Ilex_pubescens	0.004	271.516	1.58E-05	0.068	Neutral
	Vaccinium_carlesii	0.004	271.516	1.33E-05	0.051	Neutral
	Styrax_dasyanthus	0.001	271.516	3.11E-06	-0.017	Neutral
	Gardenia_jasminoides	0.000	271.516	6.29E-07	-0.033	Neutral
	Clerodendrum_cyrtophyllum	-0.001	271.516	-4.85E-06	-0.070	Neutral
	Smilax_glabra	-0.003	233.754	-1.13E-05	-0.112	Neutral
	Castanopsis_fargesii	-0.003	271.516	-1.19E-05	-0.117	Neutral
	Cyclobalanopsis_glauca	-0.003	271.516	-1.24E-05	-0.120	Neutral
	Raphiolepis_indica	-0.004	271.516	-1.32E-05	-0.125	Neutral
	Smilax_davidiana	-0.005	233.754	-1.99E-05	-0.170	Neutral
	Tarenna_mollissima	-0.006	271.516	-2.21E-05	-0.185	Neutral
	Ternstroemia_gymnanthera	-0.010	271.516	-3.75E-05	-0.287	Neutral
	Coptosapelta_diffusa	-0.011	271.516	-3.90E-05	-0.297	Neutral
	Smilax_lanceifolia	-0.009	233.754	-3.98E-05	-0.302	Neutral
	Holboellia_fargesii	-0.012	271.516	-4.28E-05	-0.322	Neutral
	Vaccinium_trichocladum	-0.012	271.516	-4.36E-05	-0.327	Neutral
	Eurya_nitida	-0.014	271.516	-5.21E-05	-0.384	Neutral
	Embelia_vestita	-0.014	271.516	-5.24E-05	-0.386	Neutral
	Itea_omeiensis	-0.016	271.516	-5.84E-05	-0.426	Neutral
	Ardisia_brevicaulis	-0.016	271.516	-6.00E-05	-0.436	Neutral
	Lithocarpus_glaber	-0.016	271.516	-6.04E-05	-0.439	Neutral
	Ardisia_crenata	-0.016	271.516	-6.05E-05	-0.439	Neutral
	Morinda_umbellata	-0.020	271.516	-7.38E-05	-0.528	Neutral

	Camellia_fraterna	-0.021	271.516	-7.83E-05	-0.558	Neutral
	Symplocos_sumuntia	-0.028	271.516	-1.03E-04	-0.725	Neutral
	Syzygium_buxifolium	-0.032	271.516	-1.18E-04	-0.819	Neutral
	Rhododendron_ovatum	-0.035	271.516	-1.31E-04	-0.906	Neutral
	Symplocos_stellaris	-0.041	271.516	-1.51E-04	-1.043	Susceptible
	Lindera_aggregata	-0.078	271.824	-2.88E-04	-1.954	Susceptible
Herb	*Hedyotis_chrysotricha*	0.123	271.516	4.52E-04	1.252	Resistant
	Woodwardia_japonica	0.016	781.405	2.09E-05	0.486	Neutral
	Dryopteris_fuscipes	-0.017	781.405	-2.21E-05	0.409	Neutral
	Carex_chinensis	-0.033	187.643	-1.74E-04	0.140	Neutral
	Dicranopteris_dichotoma	-0.504	781.405	-6.45E-04	-0.698	Neutral
	Lophatherum_gracile	-0.091	79.502	-0.001146574	-1.589	Susceptible

columns include each species' slope of IV vs. bamboo cover (β), phylogenetic distance to bamboo (MPD), calculated RI and its standardized Z-score (RI-Z), and the classification of each species as Resistant, Neutral, or Susceptible
