# Supplementary material for: The Impact of Phyllostachys heterocyclas Expansion on the Phylogenetic Diversity and Community Assembly of Subtropical Forest
Source: Plants (Basel). 2025 Oct 21;14(20):3231. doi: 10.3390/plants14203231 (PMC12566707; doi:10.3390/plants14203231)
Supplement: Supplementary file 1 [file plants-14-03231-s001.zip › Table S3 Bamboo stem proportion and stem counts of trees, shrubs, and herbs in each of the 16 study plots.pdf]

Table S3. Bamboo stem proportion and stem counts of trees, shrubs, and herbs in each of the 16 study plots.

Forest type	Plot	Bamboo proportion(%)	Stems of Moso bamboo	Stems of trees	Total stems of trees	Stems of shrubs	Stems of herbs
BF	BF1	0	0	158	158	183	15
	BF2	0	0	129	129	80	13
	BF3	0	0	114	114	155	32
	BF4	0	0	70	70	145	50
LM	LM1	28	53	134	187	204	8
	LM2	34	53	104	157	158	6
	LM3	26	43	124	167	180	7
	LM4	22	30	106	136	136	17
HM	HM1	62	109	67	176	223	11
	HM2	66	80	42	122	205	4
	HM3	61	60	38	98	245	25
	HM4	75	86	28	114	191	20
MB	MB1	100	136	0	136	397	157
	MB2	100	124	0	124	283	158
	MB3	100	117	0	117	240	101
	MB4	100	151	0	151	326	71
